# Case Report: Rare primary bronchogenic acinic cell carcinoma in a pediatric patient

**DOI:** 10.3389/fonc.2025.1640216

**Published:** 2025-10-02

**Authors:** Yuxing Sun, Jun Zhou, Jiaojiao Zhu, Ming Li, Shiwu Yang, Songtao Bin, Li Tan

**Affiliations:** ^1^ Department of Respiration, Kunming Children’s Hospital (Children’s Hospital Affiliated to Kunming Medical University), Kunming, Yunnan, China; ^2^ Department of Pathology, Kunming Children’s Hospital (Children’s Hospital Affiliated to Kunming Medical University), Kunming, Yunnan, China; ^3^ Department of Cardiothoracic surgery, Kunming Children’s Hospital (Children’s Hospital Affiliated to Kunming Medical University), Kunming, Yunnan, China

**Keywords:** acinic cell carcinoma, bronchogenic tumor, children, clinical characteristics, pathological characteristics

## Abstract

Acinic cell carcinoma (AciCC) is an uncommon tumor of the salivary glands, with primary bronchogenic cases being particularly rare, especially in children. This report presents a case of a 3-year and 11-month-old boy who exhibited symptoms of cough and fever. Contrast-enhanced CT imaging revealed a solid mass in the right upper lung lobe accompanied by atelectasis, with poor bronchial visualization and no abnormal enhancement. Bronchoscopy identified a tumor obstructing the right upper lobe bronchial orifice. Surgical resection was performed, and histopathological analysis confirmed acinic cell carcinoma without lymph node involvement. The patient remained recurrence-free during an 18-month follow-up. Pediatric primary bronchial AciCC is exceptionally rare and typically manifests with non-specific symptoms. Radiological findings such as solid lung lesions or atelectasis may lead to misdiagnosis as pneumonia. However, complete surgical excision generally yields favorable outcomes.

## 1 Introduction

Acinic cell carcinoma (AciCC) is an uncommon subtype of salivary gland tumor, accounting for approximately 25–35% of salivary gland neoplasms in pediatric patients. Among these, mucoepidermoid carcinoma remains the most frequently diagnosed subtype (1). AciCC predominantly originates in the parotid gland, representing over 90% of cases, and is generally characterized by low-grade malignancy and limited invasive potential. Distant metastases are exceptionally rare, occurring in less than 1% of patients (2). Primary bronchogenic AciCC, where the tumor arises within the bronchial tree rather than the salivary glands, is an exceedingly rare entity, particularly in children. Clinically, bronchogenic AciCC often presents with nonspecific respiratory symptoms, which can easily mimic more common pediatric conditions such as pneumonia, asthma, or inhalation of a foreign body (3). This nonspecificity frequently leads to delays in diagnosis or misdiagnosis. In this report, we describe a rare case of primary bronchogenic AciCC in a pediatric patient managed at our institution. We detail the clinical presentation, radiologic findings, and histopathological characteristics, and provide a review of the diagnostic challenges and current therapeutic approaches, as informed by the existing literature. This case underscores the importance of considering rare neoplastic causes in children presenting with persistent respiratory symptoms unresponsive to conventional treatment.

## 2 Case description

**Figure 1:**
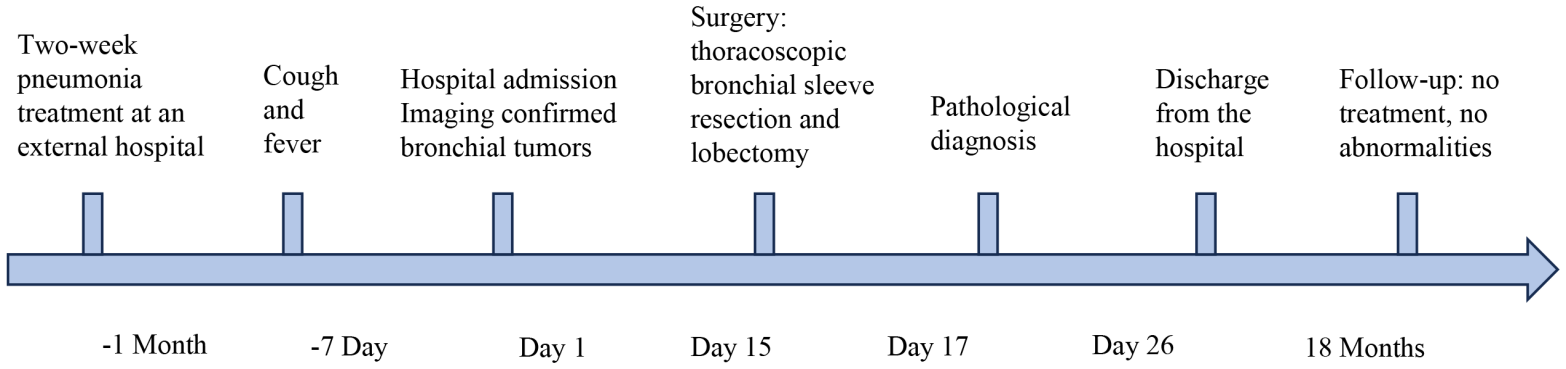
The timeline of the patient’s disease progression.

A 3-year-and-11-month-old Asian boy presented to our clinic with a one-week history of persistent, paroxysmal cough, accompanied by sputum production and intermittent fever reaching up to 39 °C. He exhibited no wheezing, shortness of breath, hemoptysis, chest pain, or dyspnea. While the fever subsided following inpatient anti-infective therapy, the cough persisted. A chest CT scan revealed bronchial obstruction, multiple exudative lesions, and partial atelectasis in the right upper lobe, prompting hospital admission for further evaluation. Notably, the patient had been hospitalized one month prior for a similar episode of cough, which resolved after a week of erythromycin therapy. He had also completed a three-day course of oral azithromycin before discharge. His birth and developmental history were unremarkable, and there was no known family history of neoplastic diseases and exposure to second hand smoke. The temporal sequence of the patient’s disease progression and clinical manifestations is described as follows (**Figure 1**).

During the pediatric assessment, the patient’s vital signs were as follows: heart rate of 117 beats per minute, respiratory rate of 32 breaths per minute, and body weight of 11.5 kg (<P3). The child appeared thin with facial flushing but exhibited no signs of lip cyanosis or the triple concave sign. Auscultation revealed rough yet symmetrical breath sounds, without any rales. The liver and spleen were of normal size, and a Bacillus Calmette–Guérin (BCG) vaccination scar was noted. Laboratory investigations showed a white blood cell count of 3.55 × 10^9^/L with a differential of 34.3% neutrophils and 52.3% lymphocytes. Red blood cell count was 5.15 × 10¹²/L, hemoglobin level was 137 g/L, and platelet count was 209 × 10^9^/L. Liver and renal function tests, cardiac enzyme levels, and serum electrolytes were all within normal ranges. Tumor markers revealed an elevated neuron-specific enolase (NSE) level of 25.12 ng/mL, while alpha-fetoprotein (AFP) and carcinoembryonic antigen (CEA) were within normal limits. Cytokine profiling showed IL-4 at 10.25 pg/mL, IL-6 at 38.7 pg/mL, and IL-17A at 24.78 pg/mL.

**Figure 2:**
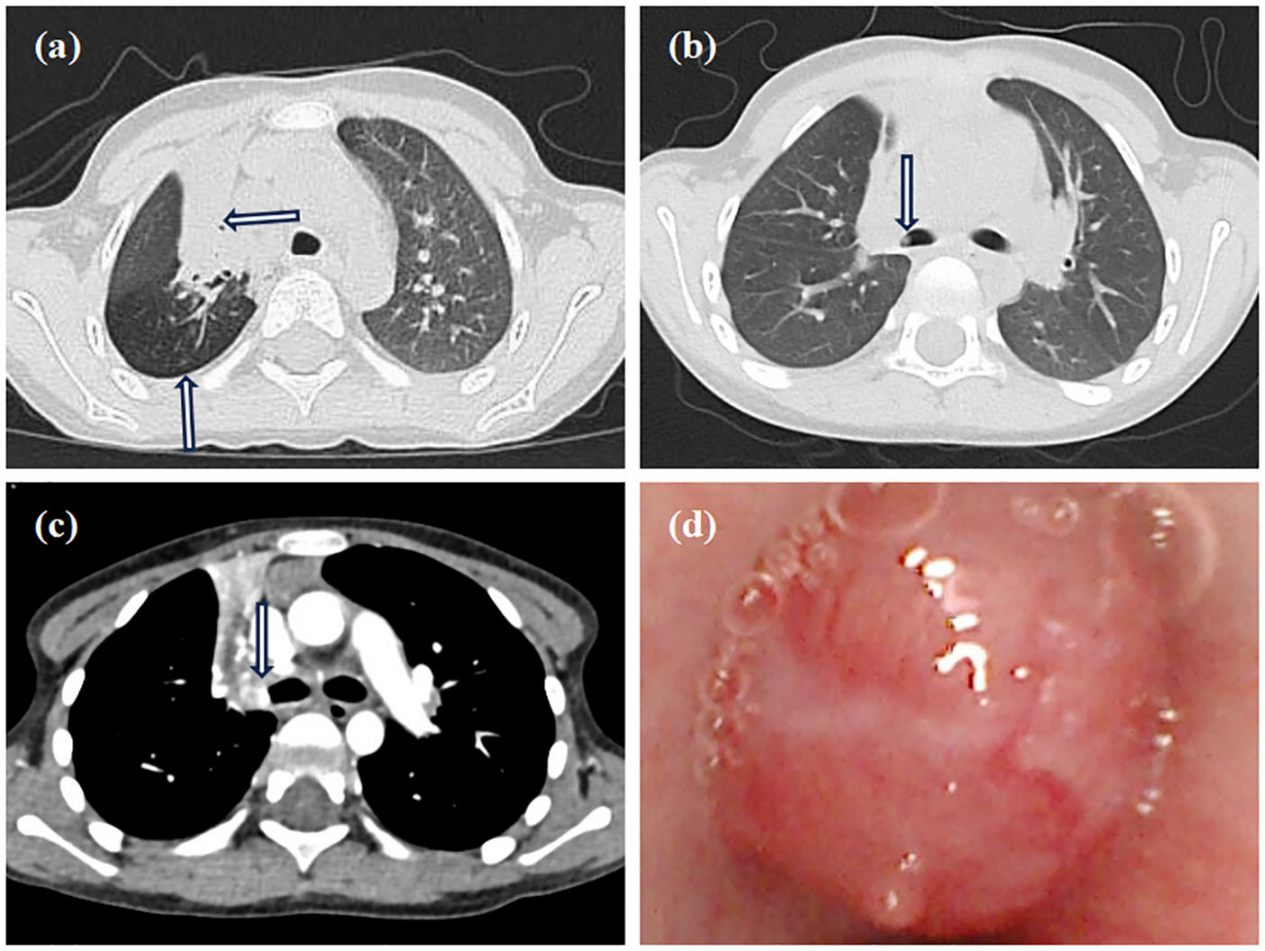
Imaging and bronchoscopy. **(a)** Chest CT shows consolidation in the right upper lobe with air trapping; **(b)** Chest CT shows a striated shadow in the right main bronchus; **(c)** The enhanced CT scan shows a bronchial cut-off sign in the right main bronchus, accompanied by heterogeneous enhancement of the adjacent tissues; **(d)** Electron bronchoscopy shows a neoplastic lesion in the right upper lobe, causing total airway obstruction with visible surface vessels.

**Figure 3:**
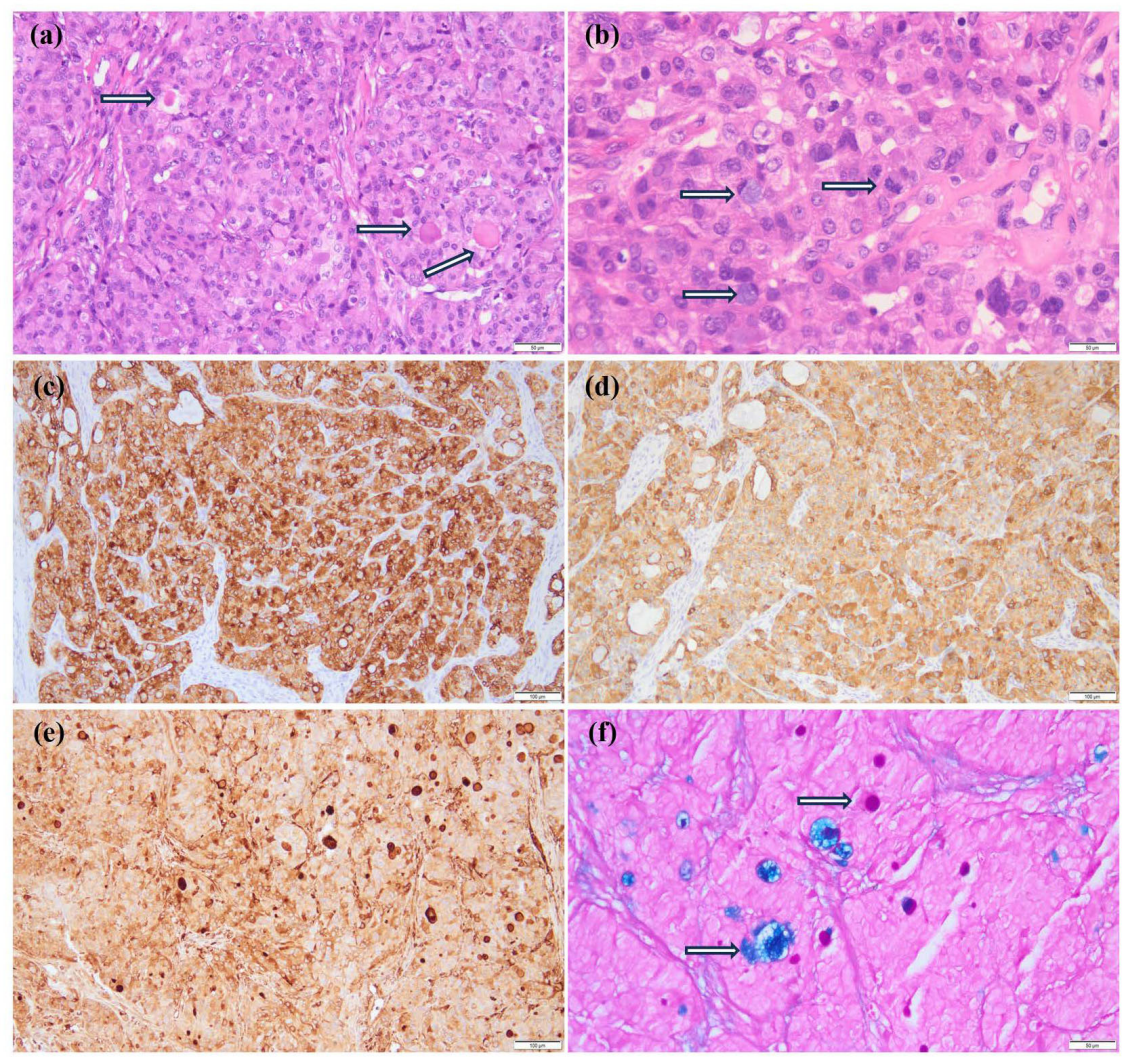
Pathological results of the patient. **(a)** The fibrous septum divides the tumor into lobules, and eosinophilic vesicles are visible (HE ×200); **(b)** Tumor cells are round, oval, or slightly polygonal with prominent nucleoli, eosinophilic granular or vacuolated cytoplasm, and evident nuclear fission (indicated by arrows). Occasionally, mucus-producing cells are present (indicated by arrows) (HE ×400). **(c-e)** Show immunohistochemical staining; **(c)** CK7 (+) (×100); **(d)** CK8/18 (+) (×100); **(e)** AACT (+) (×100); **(f)** AB/PAS staining showed that the mucous secretions of the tumor cells were blue, while the hyaline bodies were red (Special staining ×200).

Contrast-enhanced MRI of the neck, oral cavity, and maxillofacial region revealed no abnormalities. In contrast, chest-enhanced CT imaging showed solid lesions and atelectasis in the right upper lung lobe, accompanied by localized areas of increased lucency and poor bronchial visualization (**Figure 2a**). Striated soft tissue density shadows were observed in the right main bronchus (**Figure 2b**), and a bronchial cut-off sign in the right main bronchus, accompanied by heterogeneous enhancement of the adjacent tissues (**Figure 2c**). Electron bronchoscopy revealed a tumor located at the orifice of the right upper lobe, causing complete airway obstruction. A prominent vascular network was visible on the tumor surface (**Figure 2d**). The child underwent thoracoscopic bronchial sleeve resection and right upper lobectomy under general anesthesia. The resected tumor measured 0.5 × 0.4 × 0.4 cm and extended into the bronchial lumen, encapsulated by an intact fibrous capsule. Postoperative pathological examination of lung tissue was conducted. Histologically, the tumor exhibited a solid architecture with fibrous septation and featured vesicular, microcystic, and hyaline pseudocystic structures (**Figure 3a**). The tumor cells were round, oval, or mildly polygonal with prominent nucleoli. The cytoplasm was eosinophilic, granular, or vacuolated, and mitotic figures were present. Occasional mucin-secreting cells were also identified (**Figure 3b**). No residual tumor was observed at the bronchial resection margins, and all ten examined lymph nodes were free of metastasis. Immunohistochemical analysis revealed the tumor was positive for CK7 (**Figure 3c**), CK8/18 (**Figure 3d**), AACT (**Figure 3e**), and partially positive for MUC1 and MUC6. It was negative for NR4A3, DOG1, Sox10, Synaptophysin, Chromogranin A, S-100, HMB45, P63, TFE3, TTF1, CK5/6, CD56, CD34, D2-40, PAX8, and CD10. CEA was positive, and the Ki-67 proliferation index was approximately 12%. Alcian blue/Periodic acid–Schiff (AB/PAS) staining highlighted, blue-stained mucus and red hyaline material within the tumor cells (**Figure 3f**). Following consultations by multiple expert pathologists, the final pathological diagnosis was acinic cell carcinoma. No tumor involvement was identified at the bronchial surgical margins or in the lymph nodes. At the 18-month postoperative follow-up, there was no evidence of recurrence or metastasis.

## 3 Discussion

AciCC is a low-grade malignant tumor that typically arises from the salivary glands, most often affecting the parotid gland in the head and neck region. Primary occurrence of AciCC in the lungs is exceedingly rare (4). To date, most reported cases have involved adults, with only four pediatric cases described in children aged 4 to 12 years (5, 6). In this report, we present a case involving a 3-year-11-month-old boy, representing the youngest known pediatric case of pulmonary AciCC (**Table 1**). These tumors are generally well-defined and located near the bronchi, often protruding into the bronchial lumen as polyp-like masses. Clinical symptoms are primarily related to airway obstruction and may include persistent cough and hemoptysis; however, approximately one-third of cases remain asymptomatic (7). The nonspecific nature of these symptoms often leads to misdiagnoses such as pneumonia or, particularly in children, the aspiration of a bronchial foreign body. When presumed pneumonia fails to improve with standard antibiotic therapy, further diagnostic evaluation is essential to avoid delays in appropriate treatment.

**Table 1:** Clinical characteristics of primary acinic cell carcinoma of the lung in pediatric patients.

Case NO.	Age (years)	Gender	Symptoms	Site	Size (cm)	Treatment	Outcome	Reference
Case 1	12	Female	Recurrent pneumonia	Right middle lobe Right lower lobe	0.8	Bilobectomy	Alive 1 years	(6)
Case 2	4	Female	Hemoptysis night sweats	Left upper lobe	3.0	Lobectomy	Alive 2 years	(5)
Case 3	10	Female	Cough hemoptysis	Left upper lobe	1.0 1.5	Lobectomy	Alive 30 months	(6)
Case 4	8	Female	Cough hemoptysis	Left main bronchus	0.4	Bronchoscopic resection	Alive 33 months	(6)
Present Case 5	3.9	Male	Cough fever	Right upper lobe	0.5	Lobectomy	Alive 18 months	

Although clinical symptoms are often nonspecific, chest imaging can reveal indirect indicators of bronchial obstruction, such as atelectasis, air trapping, and bronchial dilation (8). In the present case, CT imaging demonstrated areas of solid consolidation and atelectasis in the right upper lobe, accompanied by air trapping. Bronchoscopy plays a crucial role in identifying congenital anomalies, airway compression, foreign bodies, and intraluminal masses, while also allowing for tissue biopsy to achieve a definitive pathological diagnosis (9).

AciCC is believed to originate either from the transformation of terminal ductal cells or from normal plasmacytoid cells. Histologically, it is categorized into four subtypes: microcystic, solid, papillary-cystic, and adenoalveolar. Bronchiolar AciCC closely resembles its counterpart in the oral cavity salivary glands. The tumor cells typically display plasmacytoid adenoid morphology, characterized by abundant cytoplasm, round or polygonal shape, granular basophilic or clear cytoplasm, small round or oval nuclei (either eccentric or central) and occasionally small nucleoli. Some cells exhibit distinctive imprinting features, while plasmacytoid follicular cells may contain granular cytoplasm and round nuclei, with hyaline material and eosinophilic granules present in some cells. Gravel bodies are observed, whereas mucinous cells are uncommon (6, 10). Immunohistochemically, acinar cells express CK (low molecular weight), α-1-antichymotrypsin (AACT), DOG1, Sox10 and amylase, and are typically PAS-positive but negative for mucicarmine staining. Notably, over 90% of AciCC cases harbor a t(4;9)(q13;q31) translocation, which leads to upregulation of NR4A3, detectable via immunohistochemistry (11). NR4A3 staining demonstrates high sensitivity and near-perfect specificity for diagnosing AciCC (12). Unfortunately, the immunohistochemical markers NR4A3 and DOG1 were not detected in this case of AciCC. This may be attributed to two potential factors. First, although the sensitivity of NR4A3 in diagnosing AciCC has been reported to range from 82% to 100%, variations in clone specificity may result in reduced sensitivity (13). Second, DOG1 demonstrates inherently lower sensitivity compared to NR4A3 in the immunohistochemical diagnosis of AciCC, with reported positivity rates of approximately 55% (14).

When evaluating bronchial AciCC in children and adolescents, several differential diagnoses should be considered: (1) Metastatic AciCC can generally be ruled out through careful clinical history and physical examination. (2) Primary lung adenocarcinoma, which typically occurs in older individuals, is characterized by the expression of TTF-1 and Napsin A; in contrast, alveolar cell carcinomas usually lack these markers. (3) Mucoepidermoid carcinoma (MEC), a malignant salivary gland tumor and the second most common primary airway neoplasm in pediatric populations, typically shows immunopositivity for P63, P40, CK7, and CK5/6, but is negative for CK20, TTF-1, and Napsin A. Additionally, the mucus-secreting cells in MEC stain positively with PAS and Mucicarmine. (4) Perivascular epithelial cell tumors (PEComas) can be distinguished by their expression of HMB45 and S-100. (5) Clear cell carcinoid tumors, though rare, are neuroendocrine in origin and typically express Synaptophysin, Chromogranin A, and CD56.

The primary treatment for AciCC is radical surgical resection, which is associated with an excellent prognosis and a 5-year survival rate exceeding 90%, although local recurrence can still occur in some cases (7). The suitability of bronchoscopic resection depends on factors such as the tumor histological subtype, size, and anatomical location (15). Some studies have demonstrated that complete tumor removal via bronchoscopy is achievable, with patients remaining free of recurrence or metastasis for up to 24 months post-procedure (16), offering a promising, minimally invasive approach that preserves airway integrity. In the present case, tracheoscopy revealed an airway-obstructing tumor with a dense vascular supply. After thorough consultation, the patient’s family opted for surgical resection instead of bronchoscopic removal. 18 months postoperatively, there was no evidence of tumor recurrence or metastasis.

## 4 Conclusion

In summary, pediatric airway AciCC is an extremely rare malignancy that can be difficult to diagnose due to its nonspecific and often misleading clinical presentation. Children may present symptoms such as chronic cough, wheezing, hemoptysis, or repeated episodes of pneumonia, which are commonly attributed to more benign conditions like asthma, infections, or inhaled foreign bodies. As a result, diagnosis is frequently delayed. It is crucial for clinicians to maintain a high index of suspicion for underlying neoplastic causes, particularly in cases where standard treatments fail to resolve symptoms or when imaging findings raise concern. Bronchoscopy plays a vital role in visualizing the lesion, and biopsy is necessary to confirm the diagnosis histologically. Once identified, the cornerstone of treatment is complete surgical resection of the tumor. Depending on its size and location, minimally invasive techniques such as thoracoscopic or endoscopic surgery may be employed to reduce surgical trauma and recovery time. In most cases, the tumor is well-circumscribed and of low-grade histology, which contributes to a generally favorable prognosis. Early detection and prompt surgical management are essential to achieving good outcomes and preventing recurrence or progression.

## Data availability

The original contributions presented in the study are included in the article/supplementary material. Further inquiries can be directed to the corresponding author.
